# Predicting climate change impacts on the distribution of endemic fish *Cyprinion muscatense* in the Arabian Peninsula

**DOI:** 10.1002/ece3.11720

**Published:** 2024-07-10

**Authors:** Ali Gholamhosseini, Masoud Yousefi, Hamid Reza Esmaeili

**Affiliations:** ^1^ Ichthyology and Molecular Systematics Research Laboratory, Department of Biology, School of Science Shiraz University Shiraz Iran; ^2^ Department of Animal Science, School of Biology Damghan University Damghan Iran; ^3^ LIB, Museum Koenig, Bonn Leibniz Institute for the Analysis of Biodiversity Change Bonn Germany

**Keywords:** Arabian Peninsula, climate change, endemic species, future climate projection

## Abstract

Freshwater fishes are facing considerable threats in the Arabian Peninsula which is considered as a highly stressed region in the Middle East. It is predicted that northern Oman is likely to face decreasing rainfall and increasing temperature in coming decades. In this study, we focused on an endemic cyprinid fish *Cyprinion muscatense*, as a model to investigate impacts of climate change on the mountain fishes inhibiting in this arid region. This species is expected to be strongly affected by climate change because of its limited distribution range in a montane area surrounded by lowlands and sea, limiting the species in shift to other areas. We used an ensemble approach by considering two regressions‐based species distribution modeling (SDM) algorithms: generalized linear models (GLM), and generalized additive models (GAM) to model the species habitat suitability and predict the impacts of climate change on the species habitat suitability. Based on the distribution models, the montane area located in northeastern Oman was identified as the most suitable habitat for this species. Our results indicate that, even under the minimum greenhouse gas emissions scenario (RCP 2.6), climate change will produce a high reduction in its potential future habitats. According to the results of percent contribution, elevation and annual minimum temperature were the most important variables in predicting the species suitable habitats. Results also showed that only a small percentage of suitable habitats for the species within boundaries of protected areas. Therefore, the impact of climate change on the species appears particularly alarming. Although our study was restricted to a single cyprinid freshwater species, decreases in potential habitats are likely predicted for other cyprinid fish species restricted to the mountains of this region, suggesting severe consideration is needed for aquatic systems in future conservation planning, especially for endemic freshwater fishes.

## Graphical abstract

We used an ensemble approach by considering two regressions based SDM algorithms to model the species habitat suitability and predict the impacts of climate change on *Cyprinion muscatense* habitat suitability. The montane area located in northeastern Oman was identified as the most suitable habitat for this species. A small percentage of suitable habitats for the species is within boundaries of protected areas.

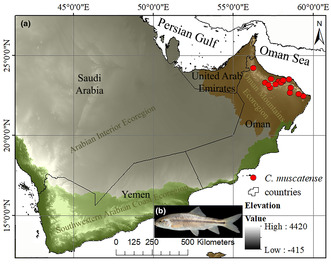

## 1 INTRODUCTION

Freshwater ecosystems are among the most threatened ecosystems on the earth (Stewart et al., 2022). Aquatic organisms in these freshwater environments face numerous threats, for example, habitat degradation, hydrology alterations, pollution, overexploitation, and invasive species (Davies & Stewart, 2013; Dudgeon et al., 2006; Reid et al., 2019; Saemi‐Komsari et al., 2023; Stewart et al., 2022). These freshwater ecosystems are considered the most vulnerable freshwater systems to climate changes and during the past several decades have faced with many treats including reduced rainfall, a decrease in the river discharge, and a warmer temperature. All of these environmental modifications have had direct or indirect negative impacts on freshwater species including fishes (Buisson et al., 2008; Harrod, 2015; Heino et al., 2009). Evidence suggests tropical and fish species in semiarid/arid ecosystems might be equally or even more vulnerable to climate changes (Korkmaz et al., 2023; Senior et al., 2019; Tewksbury et al., 2008). Climate change seems affect more freshwater ecosystems than marine ecosystems due to fragmented habitats, limited amount of water, and high sensitivity to temperature and precipitation. Effects of climate change are expected to be significant on endemic freshwater fishes with limited distribution range. If the conservation units do not contemplate current and future environmental niches, these species could become extinct.

Freshwater fishes in the Arabian Peninsula, a region located in arid and semiarid areas and considered highly stressed in the Middle East, are facing considerable threats. Oman in this region is one of most water‐stressed countries in the world. Due to factors including rapidly urbanizing populations, agricultural production that is wholly dependent on irrigation, over pumping of wells, desalinated water, and increasing groundwater salinity, water supplies and demand is not in equilibrium in the country (Al Charaabi & Al‐Yahyai, 2013). It is expected that under future climate change, balancing supply and demand will be even greater challenges. Most of Oman is expected to face an increase in future maximum temperature and decreasing rainfall in the coming decades. Therefore, groundwater recharge and surface water flow are expected to also decrease (Al Charaabi & Al‐Yahyai, 2013).

Different approaches have been implemented to evaluate the potential effects of climate change on freshwater biodiversity including fish diversity based on establishing relationships between species occurrences with environmental variables (Pacifici et al., 2015; Peterson & Soberón, 2012; Rowland et al., 2011). One of them is species distribution modeling, which is a quantitative method for understanding and projecting species distributions (Guisan et al., 2017; Guisan & Thuiller, 2005). SDMs are very useful tools to predict how species will be affected by future climatic changes (Garcia et al., 2012; Schwartz, 2012). A multitude of algorithms are available for developing species distribution models, yet the optimal algorithm remains a subject of ongoing research. Given the diversity in outcomes that different modeling methods can produce, it is advisable to employ a strategy that incorporates multiple methods (Araújo et al., 2019; Arau'jo & New, 2007). Ensemble approach integrates findings from various modeling approaches and take into account results from multiple modeling approaches (Yousefi, Heydari‐Guran, et al., 2020).

In this study, we focus on *Cyprinion muscatense* Boulenger, 1888, a fish endemic to a small montane region in the southeastern Arabian Peninsula in Oman and the United Arab Emirates. These regions are considered as highly stressed due to harsh environmental conditions and human activities that have caused multiple stressors that threaten its ecological integrity and sustainability (Esmaeili et al., 2022; Freyhof et al., 2020; Hamza & Munawar, 2009). Continued climate change is likely to severely impact in temperature and precipitation in mountain regions, especially mountains in semiarid/arid ecosystems. Therefore, poikilothermic animals like freshwater fishes are likely to be strongly affected by these changes. Using the *Cyprinion muscatense* (cyprinid fish) from mountains of the Arabian Peninsula, this paper explores how drastic these impacts can be. Information about the biology of this mountain fish is scarce beyond general observations in the original description (Esmaeili et al., 2022). We aimed to (i) model the habitat suitability of the species and identify the most important predictors of the species distribution and (ii) explore the possible effect of climate change on the distribution of this endemic fish under different climate change scenarios to serve as a model to understand impacts of climate change on the mountain fishes inhibiting in arid regions.

## 2 MATERIALS AND METHODS

### 2.1 Study area

**FIGURE 1:**
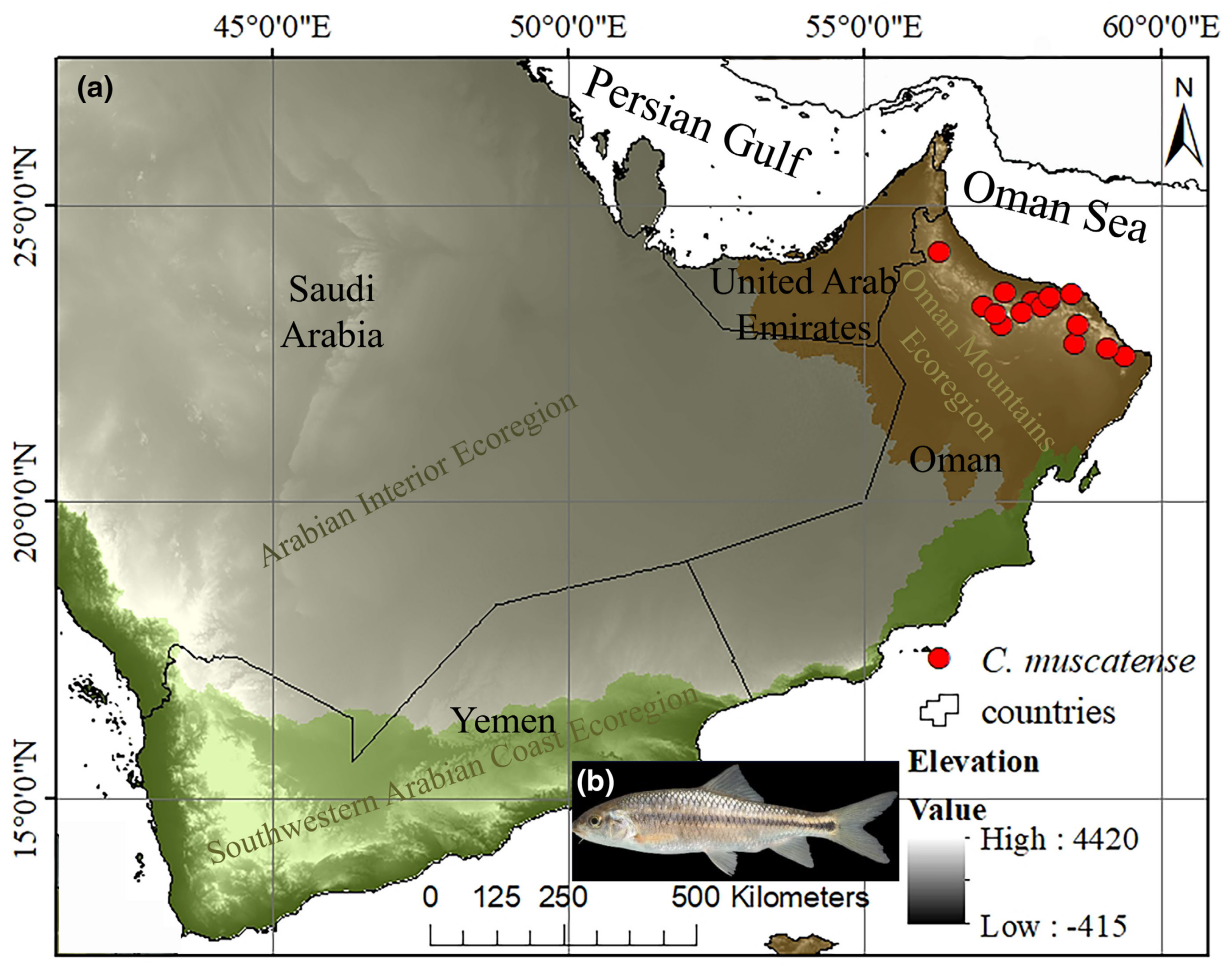
(a) Distribution map of Muscat Cyprinion (*Cyprinion muscatense*) in the Arabian Peninsula. Red circles: Distribution points, white color in background: High elevation, brown color: Oman mountains ecoregion, green color: southwestern Arabian coast ecoregion, gray color: Arabian interior ecoregion. (b) Muscat Cyprinion.

The study area in southeastern Arabian Peninsula including Oman and the United Arab Emirates encompasses three freshwater ecoregions with major habitat type of xeric freshwaters and endorheic (closed) basins: (i) Oman mountains ecoregion, which is located in the eastern part of the Arabian Peninsula, lying mostly within Oman and the United Arab Emirates, (ii) southwestern Arabian coast ecoregion which runs along the southern and western fringes of the Arabian Peninsula, bounded by the Red Sea to the west, the Gulf of Aden to the south, and the An‐Nafud and Rub' al‐Khali deserts of the Arabian interior to the east and north, and (iii) Arabian interior ecoregion, that includes the internal basins of the Arabian Peninsula (Figure 1).

### 2.2 Distribution records

The occurrence records and contemporary distribution pattern of the endemic cyprinid fish *Cyprinion muscatense* (Muscat Cyprinion) from entire drainage basins of the southeastern Arabian Peninsula including Persian Gulf, Gulf of Oman, Arabian Sea, and Rub' al Khali basins encompassing the three main ecoregions were mapped (Figure 1). Materials for this study resulted from (i) available published data (Esmaeili et al., 2022; Freyhof et al., 2020), and (ii) several extensive fieldworks that provided the geographic coordinate datasets for *C. muscatense* distribution during 2021–2022 (deposited in the Zoological Museum, Shiraz University) (Appendix A).

### 2.3 Climate and environmental variables for modeling

We downloaded freshwater environmental variables from EarthEnv databank (Domisch et al., 2015) and climatic variables from CHELSA (https://chelsa‐climate.org; Karger et al., 2017) to predict suitable habitats. As we used variables at 1 km resolution, we thinned the species distribution dataset to ensure that distribution points were at least 1 km apart.

To avoid multicollinearity and to select the most fitting predictors, we performed a VIF (variance inflation factor) test to select uncorrelated variables (VIF < 10) for modeling. This procedure yielded a list of nine bioclimatic variables including: Bio8 (mean daily mean air temperatures of the wettest quarter), Bio9 (mean daily mean air temperatures of the driest quarter), Bio11 (mean daily mean air temperatures of the coldest quarter), Bio13 (precipitation amount of the wettest month), Bio14 (precipitation amount of the driest month), Bio15 (precipitation seasonality), Bio17 (mean monthly precipitation amount of the driest quarter), Bio18 (mean monthly precipitation amount of the warmest quarter), and Bio19 (mean monthly precipitation amount of the coldest quarter). These variables can reflect the rainfall and temperature preferred by the studied fish. In addition, four freshwater‐specific environmental variables were also used in the modeling including upstream elevation, annual upstream minimum temperature, annual upstream maximum temperature, and annual upstream precipitation which describe the upstream environment in the study area.

### 2.4 Species distribution modeling

To predict the future distributions of this species under climate change scenarios, we used climatic data from two general circulation model (GCMs) for 2070 (average: 2060–2080) to identify mid‐term effects of climate change including CCSM4 and CanESM2 (average value) along with two representative concentration pathways (RCPs) including RCP 2.5 (the minimum greenhouse gas emissions scenario and less drastic climatic variation), and RCP 8.5 (the maximum greenhouse gas emissions scenario and unchecked climatic variation). A Representative Concentration Pathway (RCP) is a greenhouse gas concentration (not emissions) trajectory adopted by the Intergovernmental Panel on Climate Change (IPCC).

In this study, to model the Muscat Cyprinion habitat suitability and predict the impact of climate change on the species distribution, we used an equally weighted ensemble approach (Arau'jo & New, 2007) using two regressions based SDM methods: generalized linear models (GLM) and generalized additive models (GAM). SDMs were produced in R through the SDM package (Naimi & Araujo, 2016). Here we considered ensemble approach because different modeling methods can yield varying results, while ensemble approach allows us to simultaneously take into account results from multiple modeling approaches. In fact, ensemble approach can produce more robust models (Zurell et al., 2020).

In this study, we created 5000 pseudo‐absence data points. These were generated by randomly selecting coordinates across the study area, with the deliberate exclusion of any cells where actual occurrences of the species had been recorded. This approach ensures that pseudo‐absences are not allocated to locations where the species has been observed. Consequently, it is highly likely that the majority, if not all, of these pseudo‐absences represent areas that are unsuitable for the species. We adjusted the weight of absence records in the model algorithms to balance the presence and absence data (0.5 ratio) (Stockwell, 1999). Pseudo‐absences points were generated using the Presence Absence R package (Freeman & Moisen, 2008). To evaluate model performance, we used a split‐sample approach (75% training data and 25% evaluation data). In this study, we used area under the curve (AUC), specificity, and true skill statistic (TSS) metrics to assess the SDMs performance. These are the well‐established metrics for assessing SDMs performance in ecological studies (Guisan et al., 2017; Naimi & Araujo, 2016). Model outputs were converted into binary using the maximum training sensitivity plus specificity threshold (0.28) to calculate range changes of the species for future scenarios.

### 2.5 Protected areas coverage

To determine the representation level of present and future suitable habitats of the Muscat *Cyprinion* inside protected areas, the species habitat suitability models were overlaid on the protected areas layer. To do this, we first converted the continuous maps into binary suitable‐unsuitable maps. Then, the area of current and future suitable habitats inside protected areas was calculated using the raster package in R (Hijmans, 2017). Protected areas layers were obtained from www.protectedplanet.net.

## 3 RESULTS

The results of assessing the models' performance showed that all models performed well. The average values for the GAM for AUC, specificity, and TSS were 0.90, 0.81, and 0.78, respectively. The average values for the GLM for AUC, sensitivity, and TSS were 0.95, 0.86, and 0.86, respectively. For details, please see Appendix B. There was no significant difference in AUC between the environmental and climatic models. This suggests that both models have similar predictive performance in the context of our study.

### 3.1 Models of current and future habitat suitability

**FIGURE 2:**
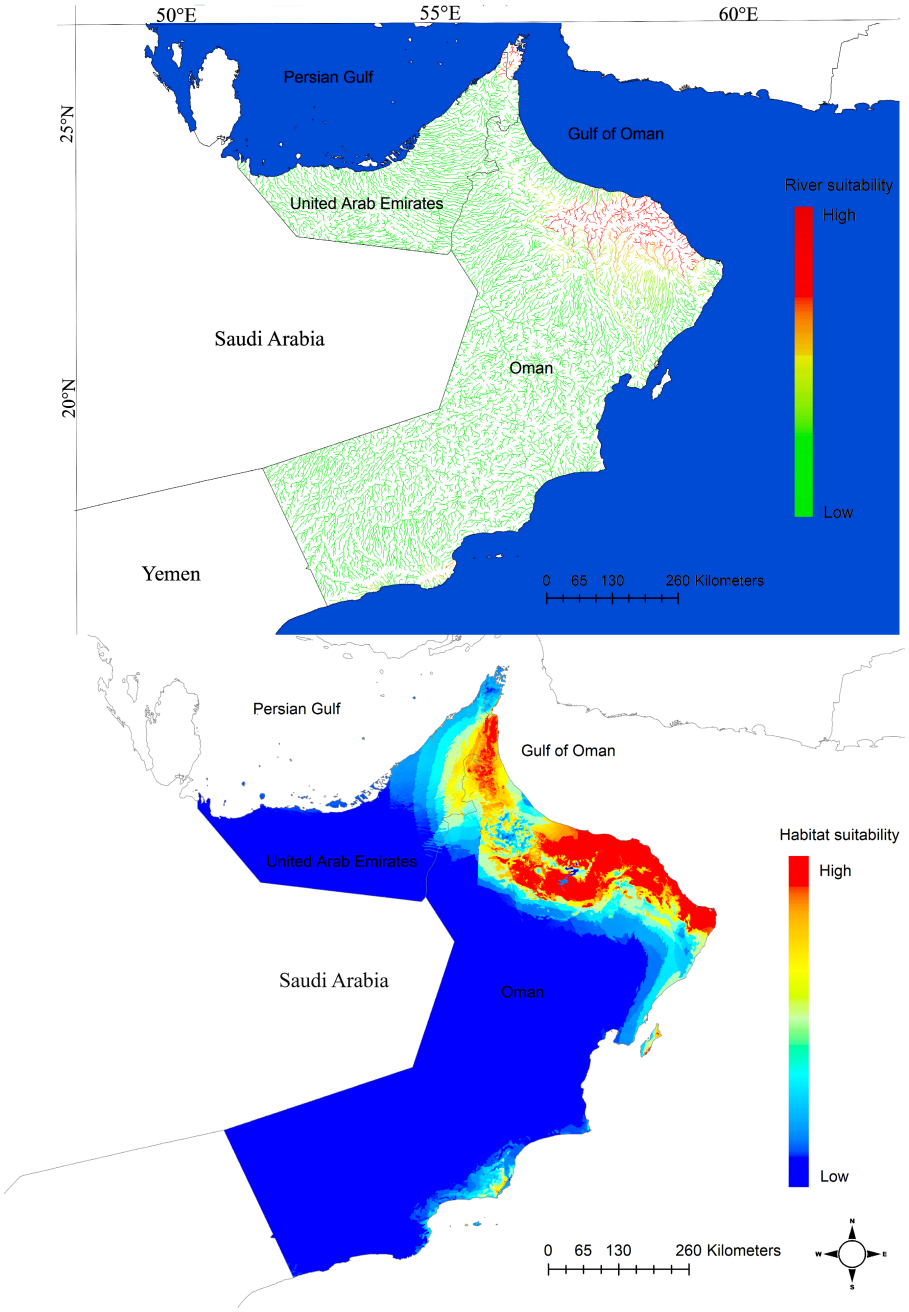
Habitat suitability of Muscat Cyprinion (*Cyprinion muscatense*) in the Arabian Peninsula based on the EarthEnv layers (upper) and climate layers (lower). The scale represents the habitat suitability index; red color indicates most suitable habitats.

**FIGURE 3:**
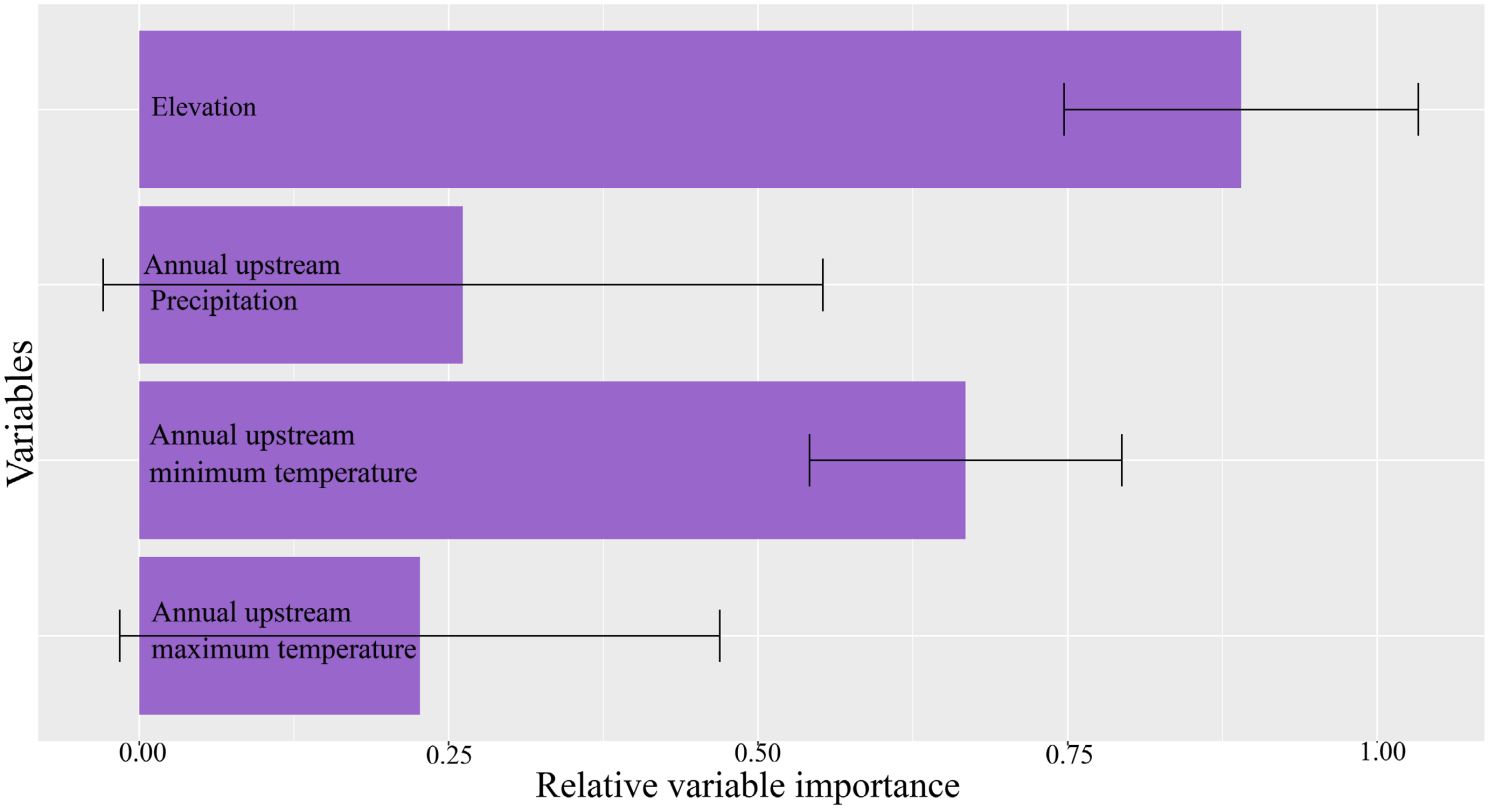
Results of relative variable importance (average and 95% CI) in habitat suitability model for Muscat Cyprinion (*Cyprinion muscatense*) based on the EarthEnv layers. The y‐axis shows the variables included in modeling and x‐axis shows the relative importance (range: 0–1).

**FIGURE 4:**
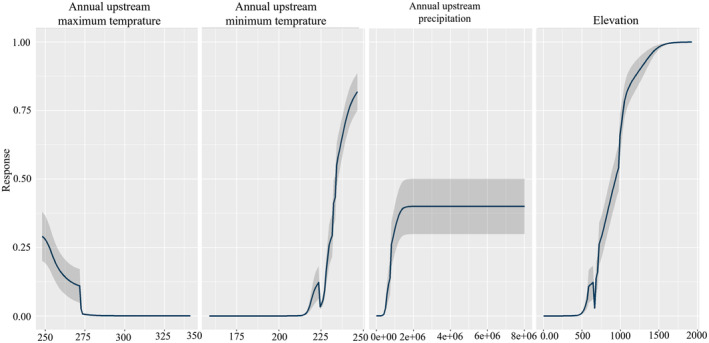
Response curves of the variables based on the EarthEnv layers for Muscat Cyprinion (*Cyprinion muscatense*) with standard errors in gray. The curves (blue lines) show how the predicted habitat suitability of the species changes as each environmental variable in the Arabian Peninsula. The y‐axis indicates the probability of presence and x‐axis shows the contribution of each variable. Temperature [°c], precipitation [mm], elevation (m).

**FIGURE 5:**
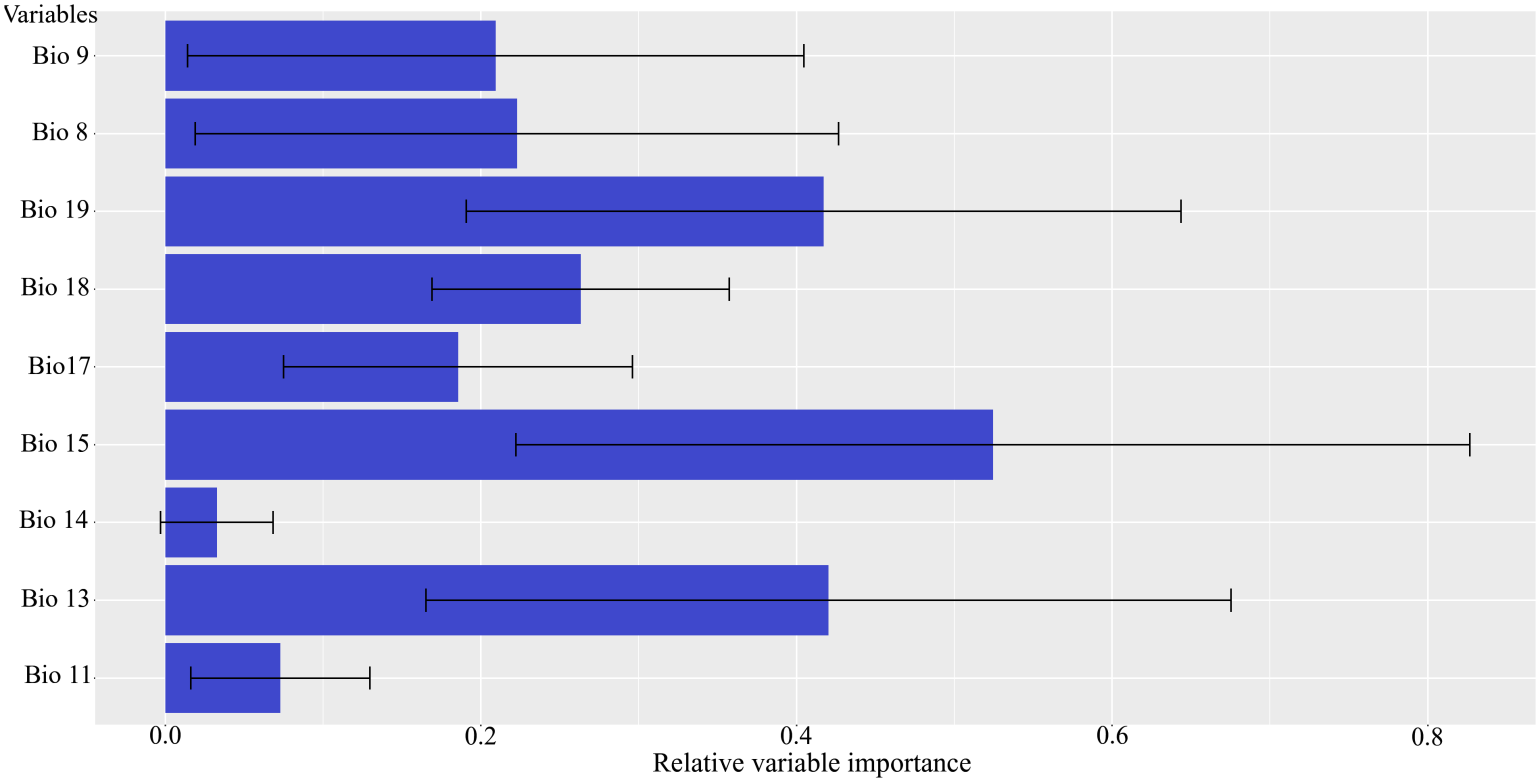
Results of relative variable importance (average and 95% CI) in climate model (ensemble model) based on the climatic layers for Muscat Cyprinion (*Cyprinion muscatense*) in the Arabian Peninsula.

The results for current habitat suitability modeling using both environmental (EarthEnv) and climatic variables are presented in Figure 2. Models predicted its distribution to the mountains of NE Oman and the United Arab Emirates in agreement with the known distribution of this endemic cyprinid fish. Contemporary models with climate variables suggested nearly similar results with the modeling using Earth‐Env variables but with more suitable habitats throughout the northwestern Oman. Results of variable importance showed the variables with the highest importance to the habitat suitability model using Earth‐Env variables were elevation with 87% contribution, followed by annual upstream minimum temperature contributing 67% (Figure 3). Based on the response curves, the species prefers elevation above 500 m above sea level and annual minimum temperature above 22°C (Figure 4). Among the climatic variables, precipitation seasonality was the most important climatic factor influencing the species distribution followed by precipitation amount of the wettest month and mean monthly precipitation amount of the coldest quarter, all related to rainfall (Figure 5).

**FIGURE 6:**
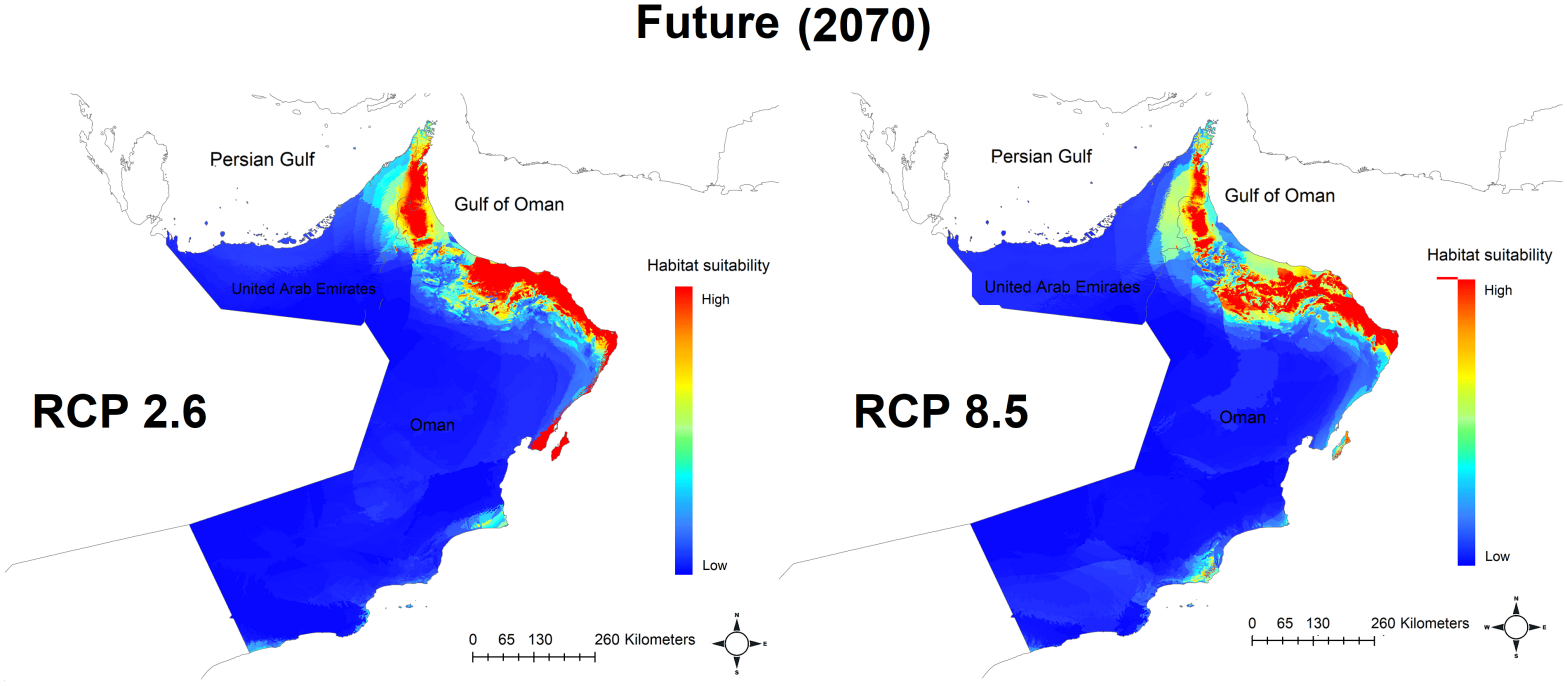
Climate suitability models of the *Cyprinion muscatense* under future (2070) climate change scenarios (RCP 2.6 in left and RCP 8.5 in right) in the Arabian Peninsula. Warmer color indicated most suitable habitats.

Climate models using climate variables of the species for future (2070) climate change scenarios are visualized in Figure 6. The models predicted suitable habitats for both future climate change scenarios mainly situated in mountains in north Oman and northeastern United Arab Emirates. To estimate the potential impact of future climate change, the predicted potential areas in the future climate scenarios were compared with the current climatic potential areas. As shown in Figure 6 and Table 1, under future climatic scenarios, the species is predicted to experience a high reduction in the potentially suitable areas. According to the table, 40,697 km^2^ of the study area is suitable for the species at present. However, under RCP2.6 and RCP8.5, this potential area is predicted to be decreased to 46% and 68% of current potential habitats, respectively. Therefore, models based on RCP8.5 showed a more severe decline in the extent of suitable habitats by 2070 (Table 1).

**TABLE 1:** Area of suitable habitat for the *Cyprinion muscatense* under current and future (2070) climate change scenarios.

Models	Area of suitable habitat (km^2^)	Status
Current	40,697	‐
Future RCP 2.6	21,789	46% decrease
Future RCP 8.5	12,841	68% decrease

### 3.2 Protected areas coverage

We estimated protected area coverage for the current and future suitable habitats of the Muscat Cyprinion. Based on the habitat suitability model, 11.7% of the species habitat is protected. According to the two climate change scenarios (RCP 2.6 and RCP 8.5), only a very small proportion (<2%) of the species' current and future suitable habitats is covered by protected areas (Table 2). The coverage to the species distribution in the current climate is better than in the future in both scenarios.

**TABLE 2:** Area of protected suitable habitats (km^2^) for the Muscat Cyprinion under current and future climate (Scenarios RCP 2.6 and RCP 8.5).

Models	Area of protected habitats (km^2^)	Percentage of suitable habitat
Habitat suitability model using environmental variables	109	11.7%
Current climate model using climate variables	407	1%
Future RCP 2.5	385	1.7%
Future RCP 8.5	186	1.4%

## 4 DISCUSSION

In this study, we modeled the habitat suitability of the endemic freshwater fish, *Cyprinion muscatense* in the eastern Arabian Peninsula with arid conditions and limited water resources using SDM package. This species is expected to be strongly affected by climate change due to its restricted distribution in mountainous areas surrounded by lowlands and sea. Our finding on loss of climatically suitable habitat align with the previous studies accessing the potential effects of climate change on freshwater ichthyofauna using SDM in some other parts of the world (e.g., Frederico et al., 2021; Makki et al., 2023; Yılmaz et al., 2021;Yousefi, Jouladeh‐Roudbar, & Kafash, 2020; Stewart et al., 2022), revealing that predicting the response of endemic elements to climate change is essential, especially for freshwater fish species that are restricted to a small geographic area and especially in areas with arid conditions.

Future modeling using climatic variables exhibits a significant decrease in the extent of potentially suitable areas for this species. Due to climate change, its distribution range may be shifted upward to a higher elevation (if possible) or reduced, or species may adapt to the new climate conditions. Although movements to a higher elevation in response to climate change are now documented for some taxa (Feldmeier et al., 2020; Freeman et al., 2018; Yousefi et al., 2015), elevation shifts may be difficult because higher elevation have more slope and lower temperature which may not be desirable for this species. Concerningly, because it faces the Oman sea in the north and east and the lowlands in the south and west, it seems the species' distribution is geographically restricted, offering limited options for dispersal.

Different taxonomic groups may respond to long‐term climate change through range shifts (Yousefi et al., 2017), reductions (Esmaeili et al., 2018; Makki et al., 2023; McMahan et al., 2020; Soliman et al., 2023), and expansions (Kafash et al., 2018; Makki et al., 2023), or by persisting in their current range (Makki et al., 2023). Shabani et al. (2018) indicated that northern Oman will remain highly suitable for Dubas bug occurrence based on four global circulation models under four representative concentration pathways (RCPs) of 2.6, 4.5, 6.0, and 8.5, for two time periods of 2050 and 2070. Similar to our findings, many studies have reported decrease in potentially suitable habitats for vertebrates, the majority for terrestrial environments (Ashrafzadeh et al., 2018; Iannella et al., 2017; Morueta‐Holme et al., 2010) and few for aquatic systems (Esmaeili et al., 2018; Kim et al., 2020; Makki et al., 2023; Yousefi et al., 2019). A decrease in the potentially suitable areas for five species belonging to *Alburnus* (Cyprinidae) from Iran was reported by Esmaeili et al. (2018) with the greatest reduction in the potentially suitable area for *A. sellal* (63.14%) and the least reduction for *A. hohenackeri* (13.65%). Makki et al. (2023) reported that 15 studied endemic fish species from Iran (N of Oman) are expected to be affected either positively or negatively by climate change (reduction for three species, expansion for five species, reduction, and expansion for seven species) during the coming decades and only one species was predicted to experience no range change. Al Charaabi and Al‐Yahyai (2013) reported that minimum temperatures will increase and experience the greatest impact from climate change in Oman based on the Intergovernmental Panel on Climate Change (IPCC) Fourth Assessment Report (AR4) A1B. Their simulation also shows that the north of Oman is expected to face decreasing rainfall in the coming decades, therefore, our studied species will face nonoptimal conditions and will lose a large area of potential suitable habitats. Although sufficient information on the physiology and ecology of this endemic species is not available, it seems decrease in rainfall in the future is likely to have significant negative impacts on freshwater fishes inhibiting in arid regions. Decreased rainfall could lead to lower water levels in rivers, reducing the available habitat for freshwater fishes and changes in water quality. Decreased rainfall could also lead to higher water temperatures in freshwater habitats.

Because of insufficient available ecological data layers for river habitats in the country, the modeling undertaken in this study lacks some factors directly related to water (e.g., river width, water depth, flow rate, hydrological, water quality variables, percentage of bed rock, boulders, rocks, cobble, gravel, sand, mud, and silt) that may affect the species distribution. Although climatic variables (air temperature and precipitation) are the primary determinants that affect species distribution (Guisan & Thuiller, 2005), incorporation above variables into climatic variables and using an ensemble model can improve the prediction of freshwater fish distribution under climate change (Kim et al., 2020). We were also aware that climate predictions are intrinsically affected by uncertainty and the effects of climate change should be mentioned with caution in the case of species that have a small number of distribution points. While the focus of the study is on climate change, other potential threats including rapidly urbanizing populations, over pumping of wells, increasing groundwater salinity, water pollution, habitat degradation, and land use change can intensify the impact of climatic factors on this freshwater fish. Despite these limitations, the study based on the all examined climatic variables predicted adverse effects in the potentially suitable habitats for this endemic species in Oman and United Arab Emirates by 2070 and likely predicted for other species restricted to mountains of these regions, suggesting serious consideration is needed for aquatic systems in future conservation planning, especially for endemic freshwater fishes.

Although freshwater ecosystems are among the most highly threatened ecosystems globally, current protected area designations often do not prioritize the requirements of freshwater organisms and has been largely based on the diversity of terrestrial organisms. The parts of these freshwater ecosystems that are located in protected areas usually occur incidental (Abell et al., 2011; Acreman et al., 2020; Heino et al., 2009). Studies report that only 5% of inland waters in Asia and Africa are encompassed by protected areas (Bastin et al., 2019). In our study, a small percentage of the distribution range of species is located in protected areas and coverage of the protected areas will decrease for the species in both future scenarios. Ecological effectiveness of protected areas can vary substantially depending on a variety of socioeconomic factors (Terraube et al., 2023). Managers of such protected areas needs consider the habitats of this species within their management planning. Conservation of the suitable habitats for this endemic cyprinid fish will yield positive outcomes for the conservation of other freshwater fish in this region. It is necessary to mention that all areas predicted as suitable for the species, and those portions that overlap with protected areas may not really be accessible to the fish. Therefore, we should be careful in mentioning the numbers, but it is clear that a small percentage of the protected areas covered the distribution range of this species, and the effectiveness of these areas for the protection also needs to be investigated.

## 5 CONCLUSIONS

Based on the distribution models, the montane area located in north Oman and northeastern of the United Arab Emirates in the Arabian Peninsula was identified as the most suitable habitat for the cyprinid fish *Cyprinion muscatense*. Our results predicted that, even under the minimum greenhouse gas emissions scenario (RCP 2.6), climate change will produce a high reduction in its potential suitable habitats. A small percentage of the distribution range of the species is located in protected areas and coverage of the protected areas will decrease for the species in future scenarios. Although our study was restricted to a single cyprinid freshwater species, we predict similar decreases in potential habitat for other cyprinid fish species restricted to the mountains of this region, suggesting urgent conservation measures are needed for aquatic systems in future conservation planning, especially for endemic freshwater fishes. While there are uncertainties associated with climate changes projections, these predictions make us aware of possible risks to consider them in environmental planning and management.

## AUTHOR CONTRIBUTIONS

**Ali Gholamhosseini:** Conceptualization (equal); investigation (equal); methodology (supporting); software (supporting); validation (equal); visualization (supporting); writing – original draft (lead); writing – review and editing (equal). **Masoud Yousefi:** Conceptualization (equal); formal analysis (lead); methodology (equal); software (lead); validation (equal); writing – review and editing (equal). **Hamid Reza Esmaeili:** Conceptualization (equal); data curation (lead); funding acquisition (lead); investigation (lead); methodology (equal); project administration (equal); supervision (lead); validation (equal); visualization (equal); writing – original draft (supporting); writing – review and editing (equal).

## FUNDING INFORMATION

Shiraz University funds the research.

## CONFLICT OF INTEREST STATEMENT

The authors declare no competing interests.

## Data Availability

Current and future climate data can be obtained from CHELSA: (https://chelsa‐climate.org) and freshwater‐specific environmental variables from the following link: https://doi.org/10.1038/sdata.2015.73. Species distribution records are presented in Appendix A. The fish specimens are available in ZM‐CBSU.

## APPENDIX A DISTRIBUTION RECORDS OF *CYPRINION MUSCATENSE* IN THE ARABIAN PENINSULA

### A.1

**Table:**

Order	Family	Genus	Species	Author	E	N
Cypriniformes	Cyprinidae	*Cyprinion*	*Cyprinion muscatense*	(Boulenger, 1888)	57.293056	22.99138889
Cypriniformes	Cyprinidae	*Cyprinion*	*Cyprinion muscatense*	(Boulenger, 1888)	56.996389	23.29305556
Cypriniformes	Cyprinidae	*Cyprinion*	*Cyprinion muscatense*	(Boulenger, 1888)	58.493611	23.50027778
Cypriniformes	Cyprinidae	*Cyprinion*	*Cyprinion muscatense*	(Boulenger 1888)	56.258611	24.20833333
Cypriniformes	Cyprinidae	*Cyprinion*	*Cyprinion muscatense*	(Boulenger, 1888)	57.343889	23.53944444
Cypriniformes	Cyprinidae	*Cyprinion*	*Cyprinion muscatense*	(Boulenger, 1888)	58.534722	22.67805556
Cypriniformes	Cyprinidae	*Cyprinion*	*Cyprinion muscatense*	(Boulenger, 1888)	57.826667	23.38083333
Cypriniformes	Cyprinidae	*Cyprinion*	*Cyprinion muscatense*	(Boulenger, 1888)	57.19	23.17
Cypriniformes	Cyprinidae	*Cyprinion*	*Cyprinion muscatense*	(Boulenger, 1888)	58.104722	23.41166667
Cypriniformes	Cyprinidae	*Cyprinion*	*Cyprinion muscatense*	(Boulenger, 1888)	56.342222	24.70777778
Cypriniformes	Cyprinidae	*Cyprinion*	*Cyprinion muscatense*	(Boulenger, 1888)	57.981944	23.29805556
Cypriniformes	Cyprinidae	*Cyprinion*	*Cyprinion muscatense*	(Boulenger, 1888)	59.3875	22.46416667
Cypriniformes	Cyprinidae	*Cyprinion*	*Cyprinion muscatense*	(Boulenger, 1888)	58.594	22.983
Cypriniformes	Cyprinidae	*Cyprinion*	*Cyprinion muscatense*	(Boulenger, 1888)	58.105833	23.45944444
Cypriniformes	Cyprinidae	*Cyprinion*	*Cyprinion muscatense*	(Boulenger, 1888)	57.647	23.18
Cypriniformes	Cyprinidae	*Cyprinion*	*Cyprinion muscatense*	(Boulenger, 1888)	59.086667	22.595278
Cypriniformes	Cyprinidae	*Cyprinion*	*Cyprinion muscatense*	(Boulenger, 1888)	56.189578	24.807644

## APPENDIX B

### B.1

Boxplot of predictive performance of species distribution models for *Cyprinion muscatense* based on mean AUC, specificity, and TSS scores for generalized linear models (GLM) and generalized additive models (GAM).
